# P072: Genetic environment and phenotypic analysis of a novel blaKPC variant produced by Klebsiella pneumonia

**DOI:** 10.1186/2047-2994-2-S1-P72

**Published:** 2013-06-20

**Authors:** J-J Mu

**Affiliations:** 1 Research and Diagnostic Center, Centers for Disease Control, Taiwan, Taipei, Taiwan, Province of China

## Introduction

A novel variant of klebsiella pneumonia carbapeneamse (KPC) was found in multidrug-resistant Klebsiella pneumonia clinical isolates from Taiwan. The novel KPC variant differs from existing KPC due to substitution at position 206 (phe→Leu). Genetic environment and phenotypes were analyzed for further understanding the novel KPC variant.

## Objectives

The aim of this study is to characterize the detailedgenetic environment of the novel bla_KPC_produced by klebsiella pneumoniaand analyze the enzymatic activity of the novel KPC variant.

## Methods

The antibiotic susceptibility of the clinical isolates and corresponding transconjugantes was determined and interpreted according to the CLSI guidelines.The plasmid carrying novel KPC variant (pKP78) was subjected into whole genome sequencing for resolving the complete sequence. The GST fusion recombinant KPC proteins were expressed for detecting the enzymatic activity.

## Results

The antibiotic susceptibilityshowed the KP producing novel KPC variant was resistant to most of the antibiotics, such as carbapenem (imipenem, ertapenem and meropenem), aztreonam, cephalosporin (cefazolin, cefotaxime and ceftazidime), but susceptible to amikacin and colistin. The whole genome sequencing has been done and resulted in 11 contigs needed to be assembled. The genetic environment surrounding novel bla_KPC_ flanked by ISKpn8 and ISKpn6-like sequences is similar with pKP048. The sequences upstream of ISKpn8 in pKP78 were, with gene order TniA transposase, IS26 transposase and partial Tn3-resolvase different from Tn3-transposase and Tn3-resolvase in pKP048. The GST-recombinant proteins were expressed and the detection of enzymatic activity is undertaken.

## Conclusion

The novel KPC variant differs from existing KPC due to substitution at position 206 (phe→Leu). The chimera of several transposon-associated elements indicated a novel genetic environment surrounding the novel bla_KPC_ gene. This residue seems not to be close to the active site. Whether it will change the activity remains unknown. The surveillance is engaging to monitor possible spreading in Taiwan.

## Disclosure of interest

None declared

